# A Centrifugal-Force-Driven Nano-Replication Strategy

**DOI:** 10.3390/nano13020346

**Published:** 2023-01-14

**Authors:** Wenning Zhao, Fan Lin, Xiuxun Han

**Affiliations:** Institute of Optoelectronic Materials and Devices, Faculty of Materials Metallurgy and Chemistry, Jiangxi University of Science and Technology, Ganzhou 341000, China

**Keywords:** nano-replication, centrifugal force, polymer, anodic aluminum oxide

## Abstract

The replication of nano-patterns is a significant means of nanomanufacturing. However, there is still a dearth of nano-replication methods that meet the requirements of both high precision and low cost. Therefore, a new strategy to achieve the replication of nano-patterns, namely centrifugal-force-driven nano-replication (CFDNR), is proposed here. An easily obtained centrifugal force which is perpendicular to the plane of a nanostructured template is designed as a driving power, to compel the dynamic polymer to fully fill the space of the template; then, the nano-pattern can be replicated on a polymer film. Anodic aluminum oxide (AAO) templates with nanohole periods of ~450 nm and ~100 nm were employed as the original masters to investigate the nano-replication behaviors. The results of morphology measurements demonstrate excellent precision. The size deviations between the nanohole in the template and the nanopillar on the polymer film are less than 4%. Furthermore, a vacuum-assisted CFDNR scheme is proposed to prevent the formation of cavitation on the polymer replica. This work provides new possibilities and choices for facile, inexpensive and high-precision nanomanufacturing.

## 1. Introduction

To date, a wide variety of ways to fabricate nanomaterials have been developed, including chemical, physical, mechanical and interdisciplinary methods, to take advantage of their enhanced properties. Nano-replication, which can make materials acquire the nanostructure from an original template, is a significant means of nanofabrication. Owing to their good plasticity, polymers are frequently employed as the replica matrix, and nano-patterned polymers have been widely applied in the fields of optics, photoelectricity, etc. [1,2,3,4,5]. At present, a number of methods, such as injection molding [6], spin coating [7], hot embossing [8] and nanoimprinting lithography [9], can realize the replication of a nano-pattern to a polymer film.

Though all of the above methods can be applied to replicate nano-patterns, the driving powers which push the dynamic polymer to fill the gaps in the nanostructured template are diverse, and the resulting replication precision, as well as the corresponding costs, are significantly different. Unquestionably, precision is an important indicator of nanofabrication level, and is of great significance to the controllability, repeatability and application performance of nano-patterns. Some methods, including injection molding, spin coating and hot embossing, have the distinct advantages of a simple process, low cost and large area. Nevertheless, limited by driving powers, these methods can make polymers acquire most of the nanofeatures of the original template, but not all, especially for nano-templates with high depth and/or low width. By contrast, nanoimprinting technology has progressed to enable high-resolution replication down to a ∼10 nm scale, and has been perceived as one of the most promising next-generation methods for nanofabrication [1,10,11]. However, the expensive equipment and the use of high-pressure gas raise the threshold for application. Hence, there is still a dearth of nano-replication methods which meet the requirements of both high precision and low cost. In essence, there may be lack of an appropriate driving power for nano-replication.

Inspired by nanoimprinting technology, we attempt to replace the gas pressure with an easily obtained centrifugal force to develop a simple, cost-effective but high-precision strategy to replicate a nano-pattern in this study. A centrifugal force which is perpendicular to the plane of a nanostructured template is designed as the driving power, to compel the dynamic polymer to fill the space of the template; then, the nano-pattern is replicated on a polymer film. This method needs neither expensive equipment nor high-pressure gas. Here, anodic aluminum oxide (AAO) served as a nanostructured template to investigate this new nano-replication method. The replication precision was evaluated using size measurements of nanoholes in the template and nanopillars on the polymer film. Furthermore, a vacuum-assisted scheme is proposed to prevent the formation of cavitation on the polymer replica. This investigation will greatly lower the threshold of nano-replication with high accuracy.

## 2. Materials and Methods

AAO templates were fabricated through a well-known two-step anodization process, as described in our previous reports [12,13]. Two kinds of AAO template with periods of ~450 nm and ~100 nm were used here.

The polymethyl methacrylate (PMMA) film was fabricated by drop casting the polymer solution with a mass concentration of 20%. Typically, 4 g PMMA (injection grade; Macklin; Shanghai, China) was added into 16 g toluene (AR; Xilong; Shantou, China) and constantly stirred for more than 12 h. Prior to use, the polymer solution was defoamed. Then, 120 µL polymer solution was dropped onto a cleaned soda-lime glass (2 × 2 cm^2^). After the solution had spread over the entire glass, the glass was put onto a hotplate and heated to 120 °C for 30 min. The PMMA thin film was obtained after being peeled from the glass.

To replicate the nano-pattern, the polymer film and template were transferred into a homemade holder, and then, vertically placed into a modified centrifugal machine, in which a heating function was added. Subsequently, they were heated to 180 °C and centrifuged with a rotation speed of 6000 rpm for 10 min. After cooling down, the centrifugation was ceased. Finally, the template was removed with NaOH (AR; Aladdin; Shanghai, China) aqueous solution, followed by rinsing with DI water and drying.

The morphological measurements of the polymer replica and template were performed using Tescan Mira3 LMH and ZEISS Sigma 300 scanning electron microscopes.

## 3. Results and Discussion

**Figure 1:**
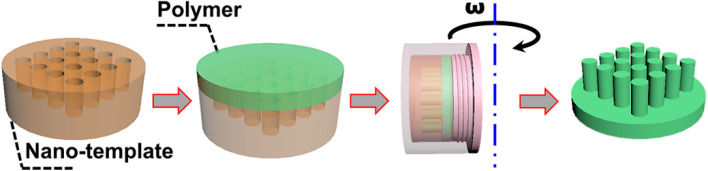
Schematic illustration of the processes of nano-replication.

Figure 1 gives a schematic illustration of the processes of centrifugal-force-driven nano-replication (CFDNR). It mainly includes three steps, that is, heating, centrifuging and demolding. In the CFDNR process, the polymer was heated to above its glass transition temperature, in order to give it mobility. Driven by the centrifugal force which is perpendicular to the plane of the nanostructured template, the dynamic polymer can vertically move into the remaining space of the template and thereby realize nano-replication. After cooling and demolding, a negative nano-pattern can be obtained. In this study, AAO is used as the template, due to the easy fabrication, low cost and highly ordered nanostructure. It is widely known that the object will gain powerful centrifugal force under the action of high-speed revolution. The magnitude of force is closely related to the rotation speed and the centrifugal radius. Additionally, the direction of force is outward, away from the rotation axis. Here, a homemade holder is used to confine the polymer film, as well as the template, and press them together tightly. In the CFDNR process, the dynamic polymer can only move towards the template and fill the nanoholes. Moreover, the loaded holder is placed parallel to the rotation axis, to make the centrifugal force perpendicular to the plane of the template and obtain a basically uniform force all over the polymer film.

**Figure 2:**
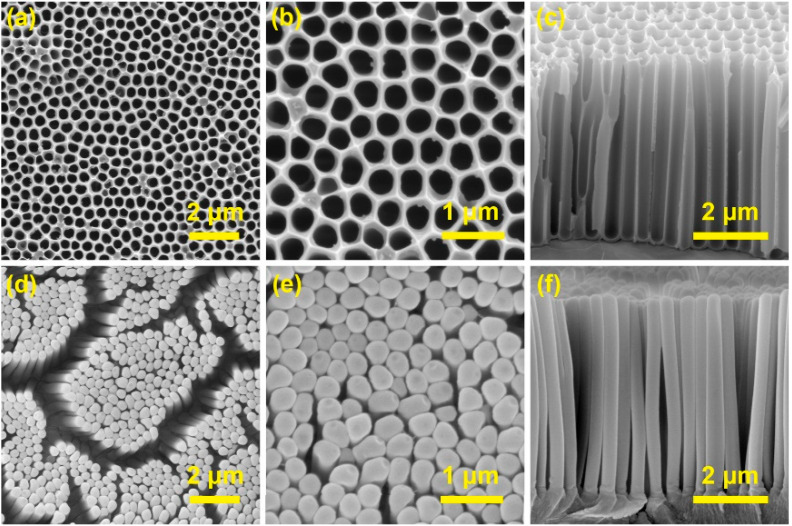
(**a**–**c**) SEM images of the AAO template. (**d**–**f**) SEM images of the polymer replica.

Figure 2 shows the representative scanning electron microscopy (SEM) images of the AAO template and the resulting polymer replica. As can be seen from Figure 2a–c, the AAO template possesses the architectural feature of a nanohole array with a period of ~450 nm. The mean diameter and depth of the nanoholes are 398 nm and 5.0 μm, respectively. Considering the rotation speed and the centrifugal radius are 6000 rpm and 4.5 cm, respectively, a centrifugal force of ~1800× *g* (gravitational acceleration) can be obtained during the CFDNR process. After the CFDNR process, the nano-pattern is negatively replicated onto the polymer film and the nanopillar array is formed, as displayed in Figure 2d–f. The mean diameter and height of the nanopillars are 395 nm and 5.2 μm, respectively, indicating that nano-replication with diameter and height deviations of less than 4% can be achieved using the CFDNR method. The heights of the polymer nanopillars are a little bigger than those of the AAO nanoholes. This swelling of the nanopillars may be attributed to the release of compressive stress during the demolding process. Obviously, if a polymer film with higher Young’s modulus is used, the swelling of nanopillars should be alleviated. In addition, the polymer nanopillars are slightly tilted and distributed in cluster form, due to the relatively high aspect ratio, the low stiffness of the PMMA material, as well as the effects of capillary force in the drying process [10,14].

**Figure 3:**
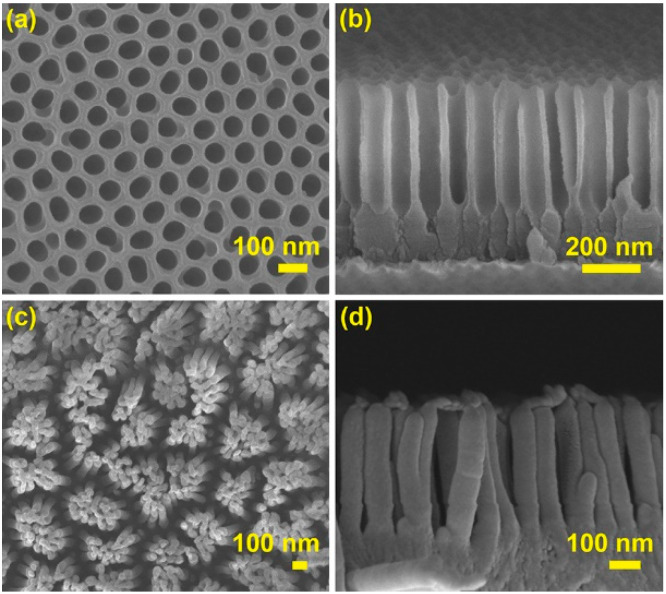
(**a**,**b**) SEM images of the AAO template with nanohole period of ∼100 nm. (**c**,**d**) SEM images of the polymer replica.

After confirming the feasibility of the CFDNR method to replicate the nano-pattern with high fidelity, to further explore the capacity of the new method, a template with a smaller nanoscale was employed. As shown in Figure 3a,b, the period of the nanohole array in this AAO template is ~100 nm, while the mean diameter and depth of the nanoholes are 76 nm and 430 nm, respectively. After the CFDNR process, the nanopillar array is well fabricated on the surface of the polymer film, as can be seen from Figure 3c,d. The mean diameter and height of the nanopillars are ~74 nm and ~433 nm, respectively. The size deviations between the nanohole in the template and the nanopillar on the polymer film are less than ~3%. In addition, the tilt of the nanopillars and the formation of the cluster occur as above.

**Figure 4:**
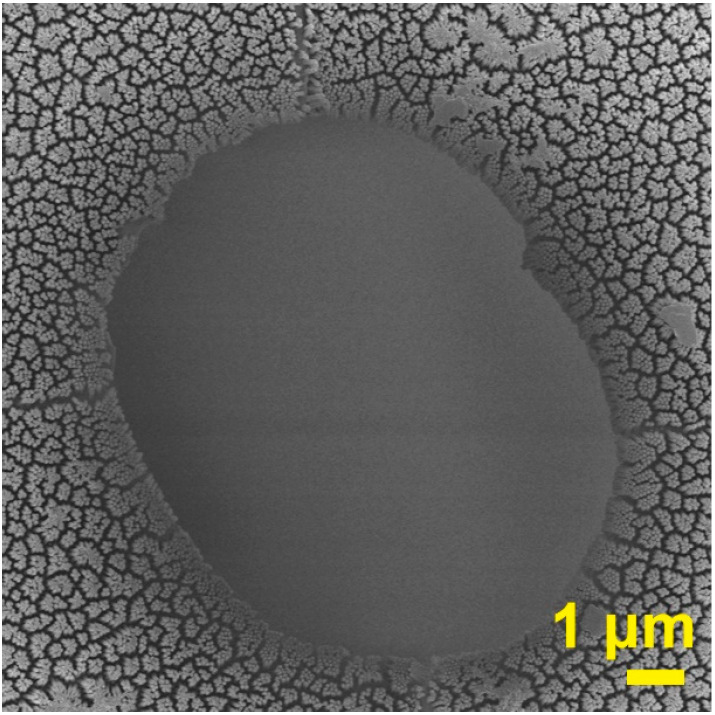
SEM image of the polymer replica with a cavitation.

Based on the above discussions, it is definite that CFDNR is a feasible and efficient way to replicate a nano-pattern onto polymer film. However, we found a problem whereby a number of cavitations existed on the polymer replica after the CFDNR process. The nanopillar is void in this cavitation region, as displayed in Figure 4. The formation of the cavitation can be ascribed to the effect of residual air during the CFDNR process. Under the action of heating, the air in the nanoholes escapes to the interface between the polymer film and the template, then, accumulates with the air in the interface gap and finally forms a series of high-pressure air regions. The high-pressure air inhibits the movement of the polymer towards the template. Undoubtedly, this is unfavorable for the fabrication of a nano-pattern with a large area. To solve this problem, a vacuum-assisted CFDNR scheme was designed and carried out. The main idea of this scheme is to eliminate the residual air in the nanoholes and the interface gap prior to the CFDNR process. The polymer film was placed onto the template and transferred into a vacuum heating system, and then, vacuumized to a pressure below 10^2^ Pa. Subsequently, the sample was heated to 180 °C for 30 min, to bring the film and template into close contact. After cooling down to room temperature, the vacuum-assisted process was completed and the CFDNR process could be conducted. The experimental results demonstrate that the vacuum-assisted CFDNR method can effectively prevent the formation of cavitations.

In this study, centrifugal force is proven to be an acceptable driving power for the replication of nano-patterns. The centrifugal force is easily acquired, and the magnitude of the force is simple to adjust. The CFDNR strategy lowers the threshold for nano-replication with high precision. Without doubt, the template and the polymer for the CFDNR method are not confined to AAO and PMMA, respectively. This method also has great potential for the fabrication of other nanomaterials and devices. Nonetheless, it is worth noting that the processing area in this work was only ~2.3 cm^2^. The processing area is a key factor for the potential application of a nanomanufacturing method. Therefore, follow-up work should focus on realizing nanofabrication with a large area.

## 4. Conclusions

In summary, to achieve the goal of nano-replication with high precision and low cost, a new method which uses perpendicular centrifugal force as the driving power is developed. In this study, two kinds of AAO serve as nanostructured templates to investigate the feasibility and capacity of the CFDNR method. The results of the morphology measurements of the polymer replica and the template demonstrate that nano-replication with size deviations of 4% and 3% was achieved for the AAO templates with periods of ~450 nm and ~100 nm, respectively. In addition, a vacuum-assisted CFDNR scheme is designed to prevent the formation of cavitation on the polymer replica. Ultimately, a simple, cost-effective and high-precision approach to nano-replication is obtained. The success of this strategy provides a new choice for nano-replication, and is of significance to the development of other nanofabrication methods.

## Data Availability

The data presented in this study are available upon request from the corresponding author.
